# LC-MS/MS Analysis of Fumonisin B1, B2, B3, and Their Hydrolyzed Metabolites in Broiler Chicken Feed and Excreta

**DOI:** 10.3390/toxins14020131

**Published:** 2022-02-09

**Authors:** Shuo Zhang, Shuang Zhou, Song Yu, Yunfeng Zhao, Yongning Wu, Aibo Wu

**Affiliations:** 1 NHC Key Laboratory of Food Safety Risk Assessment, Food Safety Research Unit (2019RU014) of Chinese Academy of Medical Science, China National Center for Food Safety Risk Assessment, Beijing 100021, China; zhangshuo6789@sina.com (S.Z.); zhaoyfl@cfsa.net.cn (Y.Z.); wuyongning@cfsa.net.cn (Y.W.); 2 SIBS-UGENT-SJTU Joint Laboratory of Mycotoxin Research, CAS Key Laboratory of Nutrition, Metabolism and Food Safety, Shanghai Institute of Nutrition and Health, University of Chinese Academy of Sciences, Chinese Academy of Sciences, Shanghai 200031, China; syu@sibs.ac.cn (S.Y.); abwu@sibs.ac.cn (A.W.)

**Keywords:** fumonisin, hydrolyzed fumonisin, broiler chicken, feed, excreta, mycotoxins

## Abstract

An accurate, reliable, and specific method was developed for the quantitative determination of fumonisins B1, B2, B3, and their hydrolyzed metabolites, HFB1, HFB2, and HFB3, in broiler chicken feed and excreta using ultra-performance liquid chromatography combined with tandem mass spectrometry (UPLC-MS/MS). The samples were extracted and diluted for the determination of parent fumonisins. Another portion of the extracted samples was alkaline-hydrolyzed and cleaned using a strong anionic exchange adsorbent (MAX) for the determination of hydrolyzed fumonisins. Chromatographic separation was performed on a CORTECS C18 column (2.1 mm × 100 mm, 1.6 μm) using 0.2% formic acid aqueous solution and methanol with 0.2% formic acid as the mobile phase under gradient elution. The six fumonisins, FB1, FB2, FB3, HFB1, HFB2, and HFB3, were analyzed by tandem mass spectrometry using multiple-reaction monitoring (MRM) mode. The six fumonisins showed good linearity, with relative coefficients of r > 0.99. The limits of quantitation (LOQs) were 160 μg/kg. At the low, medium, and high spiked levels, the recovery of fumonisins in chicken feed and excreta ranged from 82.6 to 115.8%, with a precision (RSD) of 3.9–18.9%. This method was successfully applied to investigate the migration and transformation of fumonisins in broiler chickens.

## 1. Introduction

Fumonisins are a group of toxic secondary metabolites produced by *Fusarium* and *Aspergillus* species. Among these analogues, fumonisin Bs (FBs) are the most widely distributed in animal feed and human food through contamination of grain crops [1]. According to toxicological studies, FBs have been shown to cause a variety of diseases in animals, including hepatotoxic, nephrotoxic, hepatocarcinogenic, and cytotoxic effects in mammals [2]. Epidemiological investigations have shown that in high-exposure populations, fumonisins are associated with esophageal cancer, primary liver cancer, neural-tube defects, and cardiovascular diseases [3]. The International Agency for Research on Cancer (IARC) classifies fumonisins as category 2B carcinogens (probable human carcinogens) [4]. To protect human health from the risk of FBs, the Joint FAO/WHO Expert Committee on Food Additives (JECFA) set a provisional maximum tolerable daily intake (PMTDI) for the group of fumonisins (B1 and its analogues B2 and B3) at 2 µg/kg body weight per day [5]. Numerous countries have issued maximum levels for fumonisins in food and feed [6,7,8].

**Figure 1:**
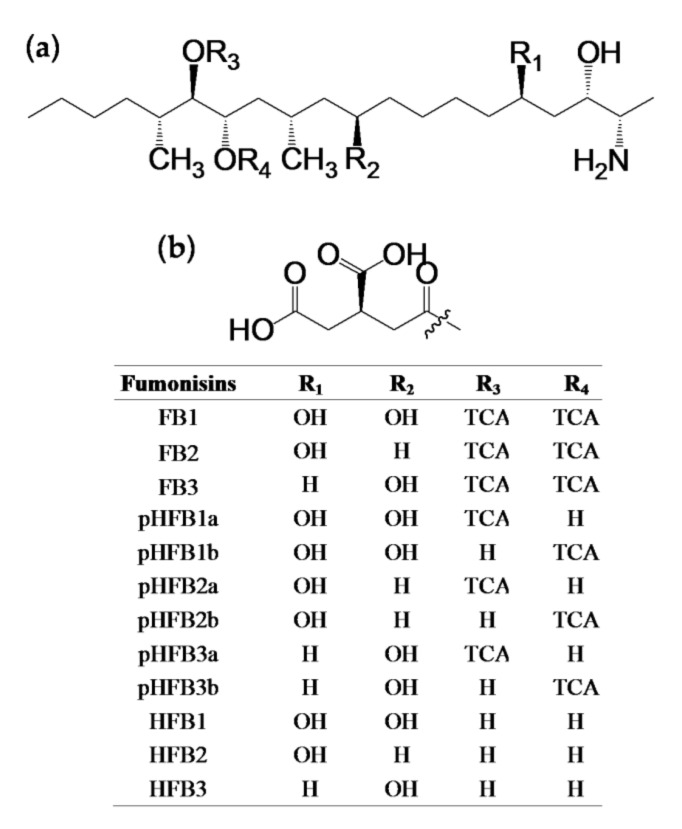
Structures of fumonisin analogues, FBs (FB1, FB2, and FB3), pHFBs (pHFB1a, pHFB1b, pHFB2a, pHFB2b, pHFB3a, and pHFB3b), and HFBs (HFB1, HFB2, and HFB3). (**a**) Structures of fumonisin backbones; (**b**) structure of tricarballylic-acid side chain (TCA).

Hydrolyzed and partially hydrolyzed fumonisins originate from FBs by hydrolysis of tricarballylic acid (TCA) groups, which are replaced by hydroxyl groups (Figure 1). Hydrolyzed and partially hydrolyzed fumonisins can occur in feed processing, and endogenous hydrolysis products of FBs occur in the metabolic pathways of animals [9]. The metabolism and biotransformation of FBs and HFBs through the intestinal microbiota and liver have been studied extensively in pigs. [10,11]. Research on the metabolites of FBs and HFBs in chicken excreta, on the other hand, is very limited and worthy of further study. Moreover, FBs and their hydrolyzed forms in broiler chicken excreta are perspective exposure biomarkers for the assessment of feed exposure, fumonisin risk, and feed carryover to poultry products. Furthermore, these biomarkers can be applied to monitor fumonisins in the environment, as well as the living conditions of broiler chickens.

In order to further our understanding of the biotransformation of FB1 and its in vivo metabolites in avian species, as well as to validate these potential exposure biomarkers, reliable and sensitive analytical methods are needed to identify and quantify FBs and HFBs in chicken feed and excreta. 

Up to now, some animal studies have adopted a similar strategy to simultaneously quantify individual FB1, HFB1, and pHFB1a+b using [13C]-FB1, [13C]-HFB1, and [13C]-pHFB1a+b as isotope internal standards [12,13,14]. However, these pHFB and [13C]-hydrolyzed fumonisin standards are commercially unavailable. 

Dall’Asta’s method involves alkaline hydrolysis during the extraction step [15], during which free, partially hydrolyzed, and matrix-bound fumonisins, as well as some modified fumonisins (e.g., O-fatty acyl FB), are transformed into hydrolyzed fumonisins. Then, the hydrolyzed fumonisins can be determined and expressed as total fumonisins. Although this method has been more widely used in maize and maize-based products to measure masked fumonisins [16,17,18], we optimized this alkaline-hydrolysis preparation procedure and developed an LC-MS/MS method to determine FB1, FB2, FB3, and their hydrolyzed metabolites in broiler chicken excreta and feed. Moreover, in our study, HFBs and their corresponding [13C]-internal standards were prepared using the MAX solid-phase-extraction column-purification method after alkaline hydrolysis.

## 2. Results and Discussion

### 2.1. Sample Preparation 

Homogeneous feed or excreta samples were subjected to ACN/water/formic acid (74/25/1, *v*/*v*/*v*) solvent according to Hahn’s method [12]. With little modification to the shaking and ultrasonic steps, the extraction efficiency of all fumonisins (FB1, FB2, and FB3) was found to be satisfied (>90%). A portion of the extract was diluted and added to 13C-FBs standards for FBs analysis. Another portion of the extract was added to 13C-FB standards and subjected to alkaline hydrolysis so that the fumonisins, partial hydrolyzed fumonisins, and other conjugated metabolites could be converted to HFBs. Thus, the total amount of metabolites could be obtained from their difference.

#### 2.1.1. Alkaline Hydrolysis

**Figure 2:**
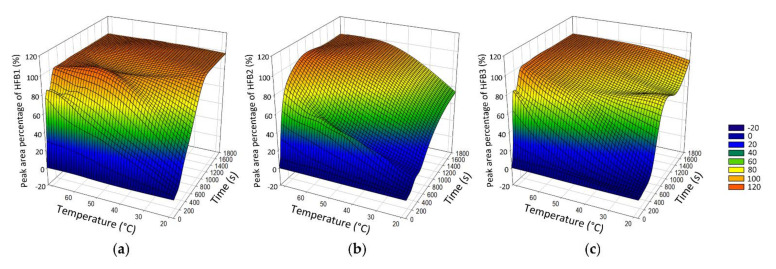
Surface graph of effects of temperature and time on the hydrolyzation of fumonisin B1 (**a**), B2 (**b**), and B3 (**c**).

Complete hydrolysis of FBs into HFB is crucial to ensure the accuracy of the calculation. Therefore, in this study, we monitored the hydrolysis reaction for individual FBs under different conditions. LC-MS/MS chromatograms of the reactant (FBs), intermediate product (pHFBs), and final product (HFBs) in the hydrolysis reaction are shown in Figure S1. We observed the peak-area percentages of individual HFBs among all FBs, pHFBs, and HFBs while hydrolyzing 1.0 µg/g individual FBs at different temperatures (25–70 °C) and different reaction times (0–7 h). The reaction process is shown in Figure 2.

The results indicate that after reacting in a water bath at 70 °C for 10 min, all FBs can be converted into HFBs. In the actual sample detection, taking into account factors such as sample matrix and concentration variation, the conditions of the hydrolysis reaction were set to react in a water bath at 70 °C for 1 h.

#### 2.1.2. MAX Procedure

In the existing methods [15,16,17,18], after hydrolysis, the hydrolyzed fumonisin is extracted with ethyl acetate or acetonitrile, then concentrated and reconstituted for analysis. This process requires several extraction steps and combination of the solvent from each step into a large volume of ethyl acetate. It then takes time to evaporate all the solvents to dryness. To simplify this procedure, we utilized a MAX solid-phase extraction column. Oasis MAX is a mixed-mode polymeric sorbent with anion-exchange groups that can achieve high selectivity for extraction of target compounds by adjusting the pH value and polarity of the solvent. The MAX sorbent was preconditioned with methanol and distilled water. Then, the hydrolyzed solution was loaded onto an Oasis MAX cartridge. The sorbent was washed with 2% ammonium hydroxide, hence the alkaline hydrophilic compounds was removed. FBs, pHFBs, and HFBs (partially lost or totally lost tricarballylic acid (TCA) groups of FBs) were able to be retained on the MAX SPE column. Then, the MAX sorbent was eluted with acid methanol so the acidic and relatively weak polar compounds, such as FBs, pHFBs, and HFBs, could be selectively released from the sorbent. It is easier to achieve standard curve preparation sample batch processing with the same method, lowering the cost of solvent and time. 

**Figure 3:**
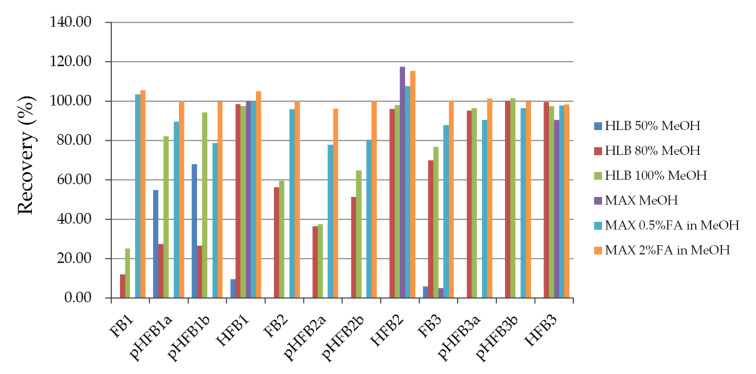
Recoveries of hydrolyzed fumonisins under different cleanup methods (*n* = 3).

We compared two types of SPE columns: HLB and MAX. MAX showed the best purification efficiency (Figure 3). The ionized impurities formed during hydrolysis could not be retained in the strong anion-exchange column and were removed during the washing process, then condensed and eluted with 2% formic acid methanol. 

### 2.2. Preparation of Standard Solutions

Free fumonisins FB1, FB2, FB3, and their 13C-isotope internal standards are commercially available. However, hydrolyzed fumonisins HFB2, HFB3, and their 13C-isotope internal standards, ^13^C_34_-HFB2 and ^13^C_34_-HFB3, are not commercially available. In several reports, HFB2 and HFB3 were synthesized one by one from FB2 and FB3, according to published literature, and further prepared as standard work solutions. In this study, HFB standard curves containing their matching ^13^C-HFBs were prepared from the FB standard curves according to the same hydrolysis procedure mentioned in Section 2.1.1. As a result, [13C]-FBs and FBs in the standard curves were completely transformed to the corresponding [13C]-HFBs and HFBs. Meanwhile, the problem of the lack of commercially available [13C]-HFB and HFB standards can be solved. Furthermore, the sample and standard curve can be prepared in the same batch for HFBs analysis.

### 2.3. Optimization of LC-MS/MS

**Figure 4:**
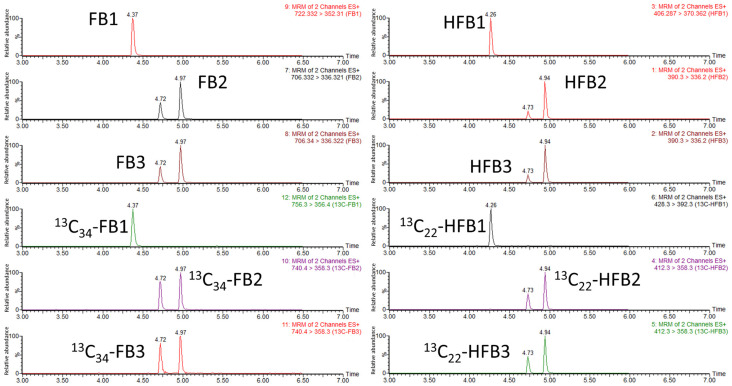
LC-MS/MS chromatograms of FBs, HFBs, and their 13C internal standards.

Among the six analytes, FB2 and FB3 are isomers, sharing the same precursor/product ion pair, as is the case for HFB2 and HFB3. In order to avoid interference in the same monitor channel, it is necessary to optimize the mobile phase gradient and select an appropriate chromatographic column to achieve baseline separation. We investigated two chromatographic columns, ACQUITY UPLC BEH C18 (2.1 mm × 100 mm, 1.7 µm) and CORTECS C18 (2.1 mm × 100 mm, 1.6 µm), in two mobile phase systems: 0.1% formic acid water-acetonitrile and 0.1% formic acid water-methanol. The CORTECS C18 column (2.1 mm × 100 mm, 1.6 µm) with 0.1% formic acid-methanol had a higher response on all six analytes but with tailing peaks of FB2, FB3, HFB2, and HFB3. With the increase in the proportion of formic acid and adjustment of the elution gradient, the symmetrical peaks of all the analytes with baseline separation were obtained in the mobile phase of the 0.2% formic acid water-0.2% formic acid methanol system. Moreover, chromatographic separation was achieved within a short run time of 10 min. Final liquid chromatography conditions and the extracted ion chromatograms of FB1, FB2, FB3, HFB1, HFB2, HFB3, and their corresponding stable isotope internal standards are shown in Section 4.4, and Figure 4.

### 2.4. Method Validation

#### 2.4.1. Linearity and Sensitivity

Table 1 shows that six analytes, FB1, FB2, FB3, HFB1, HFB2, and HFB3, have good linearity, with correlation coefficients of r > 0.99 in their linear ranges. Different amounts of FB1, FB2, and FB3 standards were spiked into the blank feed and excreta matrix, respectively. These spiked samples were hydrolyzed and analyzed according to Section 4. The limits of detection (LODs) and limits of quantification (LOQs) were set based on the signal-to-noise (S/N) ratios of the spiked blank matrix. The LODs for analytes were 5 µg/kg in feed and excreta. Considering the water weight loss in dry samples from other reports and samples without drying in our study, the sensitivity of our method was found to be in the same range as that reported by Grenier et al. [13] and Hahn et al. [12]. The sensitivities of all the analytes in feed and excreta were high enough to allow for accurate quantification of FBs and HFBs in chicken feed and excreta samples. LOQs were much lower than CAC and the EU maximum level of 200–4000 µg/kg for the sum of FB1, and FB2 [6,19]. Generally, the levels of FBs and their metabolites in animal feed and feces are much higher than those in animal tissue, blood, and urine. Accordingly, large differences in dilution factors are used in methods for different matrices to fit the linear ranges of the analytes. As a result, LOQs of methods for animal feces are in the range of 160–940 µg/kg, normally with a dilution factor of 250–800, while LOQs of the methods for animal urine are at levels of 940 µg/kg FB1, with a dilution factor of 0.9 [12]. For a method of testing animal blood with good phospholipid removal efficiency of Ostro 96-well plates and a dilution factor of 4, the LOQs of FB1, FB2, and HFB1 can be lowered to 1–2.5 ng/mL in broiler chicken plasma [14].

#### 2.4.2. Matrix Effects

The results for matrix effects (ME) are also shown in Table 1. For FBs, the dilute-and-shoot strategy resulted in a moderate signal enhancement (ME of 102.5–137.6%) in excreta and signal suppression (ME of 76.5–112.3%) in feed. For HFBs with MAX purification, matrix effects were improved to 84.6–115.4% in feed and excreta. In our method, matrix effects were further minimized by using isotope-labeled internal standards.

#### 2.4.3. Accuracy and Precision

Accuracy and precision were tested at three different spiked levels (low, medium, and high). The recoveries were between 82.6 and 115.8%, and the relative standard deviations were between 3.9 and 18.9%. The acceptable ranges were met for all analytes in both the feed and excreta matrices. The results can be found in Table 2.

### 2.5. Analysis of Real Samples

**Figure 5:**
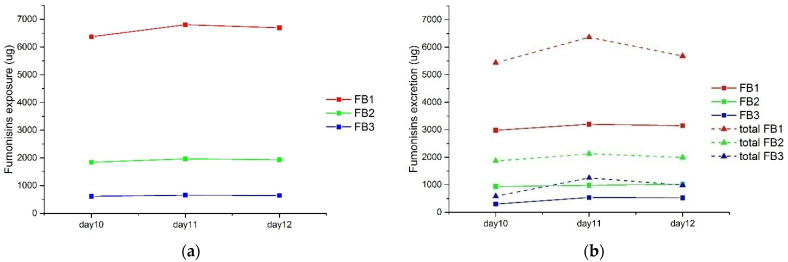
Fumonisin exposure (**a**) and excretion (**b**) in a broiler chicken study over three consecutive days.

The applicability of this developed UPLC-MS/MS method was tested with real broiler chicken feed and excreta samples. These real samples were received from a two-week FB exposure study. The feed sample was taken from an FB-contaminated diet. The excreta samples were taken from 12 chickens that received this diet for 12 days. The detailed information on the feeding trail is described in Section 4.2. The concentrations of FBs in feed samples were determined to be 10.8 mg/kg FB1, 3.12 mg/kg FB2, and 1.04 mg/kg FB3. HFBs in the feed sample without hydrolysis were also analyzed, with no HFB1, HFB2, or HFB3 detected. The exposure levels for day 10, day 11, and day 12 are presented in Figure 5a. For excreta samples, the levels of FBs and HFBs were analyzed both before and after hydrolyzation. LC-MS/MS chromatograms of analytes in a real sample are presented in Figure S2. The amounts of FB1, FB2, and FB3 before hydrolyzation are shown as solid lines in Figure 5b. HFB1, HFB2, and HFB3 after hydrolyzation are expressed as total FB1, FB2, and FB3, respectively, to represent the sum of fumonisins and their metabolites. Between the 10th and 12th day of feeding, as shown in Figure 5b, the vast majority of exposed FBs (approximately 90%) were excreted with excreta. The excretion of parental fumonisins was at approximately half of their total fumonisin levels. These results imply that the other half of the fumoninsins could have been metabolized into modified fumonisins in avian species, such as pHFBs, N-acyl-FBs, and/or N-acyl-HFBs. These modified forms of fumonisins can be transformed into HFBs under alkaline hydrolysis. Although very little is known about the metabolism of fumonisins in animals [20] and most fumonisin metabolites can still not be identified and determined directly, with our method, the total amount of these metabolites can be quantified. Therefore, the developed method is applicable in fumonisin-exposure investigations in avian species to better understand the biotransformation of FBs. More detailed data from the animal study will be presented in our forthcoming results.

## 3. Conclusions

An LC-MS/MS method was established for the quantitative determination of FBs (FB1, FB2, FB3) and their metabolites that can be converted into hydrolyzed fumonisins (HFB1, HFB2, HFB3) by alkaline hydrolysis in broiler chicken feed and excreta. Furthermore, the sample-preparation procedure and LC-MS/MS conditions were optimized. The commercially unavailable standards can be prepared using the same procedure used for sample preparation. The method was validated for all analytes in broiler chicken feed and excreta, with good validation results in terms of linearity, sensitivity, precision, and accuracy. The method was successfully applied to the analysis of real samples from an exposure study.

## 4. Materials and Methods

### 4.1. Chemicals and Reagents

Acetonitrile, methanol, and formic acid were of LC/MS grade (Fisher Scientific, Loughborough, UK). All other chemicals were of analytical grade or better. Deionized water (18.2 MΩ cm) was collected from a Milli-Q system (Millipore Corp., Bedford, MA, USA). Oasis MAX 3cc 60 mg cartridges and Oasis HLB 3cc 60 mg cartridges were purchased from Waters (Milford, MA, USA). Standard solutions of mixed FB1, FB2, and FB3 with certified concentrations of each analyte were purchased from Biopure (Tulln, Austria). [^13^C_34_]-FB1, [^13^C_34_]-FB2, and [^13^C_34_]-FB3 standard solutions with certified concentrations were also purchased from Biopure (Tulln, Austria).

### 4.2. Sample Collection

All samples were obtained from a broiler chicken two-week fumonisin exposure study. Twelve 7-day-old male Ross 308 broiler chickens were given an FB-contaminated diet for two weeks. The FB-incurred feed was prepared, and the levels of 6 analytes were measured. The remaining feed was weighed every 24 h. Thus, the consumption of feed for 12 chickens could be calculated. Every 24 h, excreta samples of the 12 broiler chickens were pooled, weighed, and homogenized. All samples were stored at −20 °C until further analysis. The animal experiment was approved by the Ethics Committee of Shanghai Veterinary Research Institute, Chinese Academy of Agricultural Sciences (project identification code: SV-20200906-Y06) on 6 September 2020.

### 4.3. Sample Preparation

A total of 5 g of feed sample or excreta sample was homogenized in 20 mL of ACN/water/formic acid (74/25/1, *v/v/v*). Samples were then shaken for 30 min and ultrasonicated for another 30 min. After centrifugation, 50 µL of supernatant (15 min at 8000 rpm) was combined and vortexed with 950 µL of distilled water. Then, a 50 μL aliquot of the resulting solution was mixed with 10 μL 13C-FBs IS working solution and diluted with 850 µL of 0.2% FA in methanol/water (1:9) prior to LC-MS/MS injection for FBs analysis. 

Another 50 µL aliquot was added with 10 μL of ^13^C-FBs IS working solution and mixed with 850 µL of 2.5 M NaOH aqueous solution. After hydrolysis for 2 h at 70 °C, the samples were cooled down to ambient temperature and added to 2 mL of water. Then, the hydrolyzed solution was loaded onto an Oasis MAX cartridge. The sorbent was washed with 3 mL of 2% ammonium hydroxide and eluted with 3 mL of 2% formic acid in methanol. Finally, the eluent was evaporated under nitrogen to dryness and reconstituted with 0.2% FA in methanol/water (1:9) prior to LC-MS/MS injection for HFBs analysis.

### 4.4. LC-MS/MS

LC–MS/MS analysis was performed on an ultra-high-performance liquid chromatography system (Acquity UPLC H-Class, Waters) coupled to a tandem mass spectrometer (Exvo TQS, Waters) in multiple-reaction monitoring (MRM) mode with an electrospray ionization source in positive mode (ESI+). Chromatographic separation was achieved on a CORTEX C18 column (10 mm × 4.6 mm, 5 μm). The temperatures of the column oven and autosampler tray were set at 40 °C and 10 °C, respectively. The flow rate was 0.4 mL/min. Mobile phase A consisted of 0.2% FA in water, whereas mobile phase B consisted of 0.2% FA in methanol. Gradient elution was performed: 0 min (90% A, 10% B), 0–6 min (linear gradient to 90% B), 6–8 min (10% A, 90% B), 8–8.1 min (linear gradient to 90% A), 8.1–10.0 min (90% A, 10% B). The following mass spectrometry parameters were used: capillary voltage: 2.5 kV; desolvation temperature: 500 °C; desolvation gas flow: 800 L/h; collision gas: 0.15 mL/min. The MRM transitions that were monitored for analytes are shown in Table 3.

## Figures and Tables

**Table 1:** Linearity (linear range and correlation coefficients), sensitivity (LODs and LOQs) of six fumonisins in different matrices.

Analytes	Linear Range (ng/mL)	Correlation Coefficients r	Chicken Feed			Chicken Excreta		
			LOD (µg/kg)	LOQ (µg/kg)	ME %	LOD (µg/kg)	LOQ (µg/kg)	ME %
FB1	0.5–1000	0.9995	50.0	160	112.3	50.0	160	137.6
FB2	0.5–1000	0.9975	50.0	160	86.5	50.0	160	120.2
FB3	0.5–1000	0.9947	50.0	160	76.5	50.0	160	102.5
HFB1	0.5–1000	0.9991	50.0	160	95.6	50.0	160	115.4
HFB2	0.5–1000	0.9979	50.0	160	105.6	50.0	160	89.5
HFB3	0.5–1000	0.9968	50.0	160	84.6	50.0	160	92.7

**Table 2:** Recoveries and precision of fumonisins in broiler chicken feed and excreta (*n* = 6).

Analytes	Spiked Levels (µg/kg)	Chicken Feed		Chicken Excreta	
		Recovery%	RSD%	Recovery%	RSD%
FB1	200	89.6	7.5	90.9	3.9
	4000	95.6	4.5	102.5	10.6
	20,000	90.7	6.6	105.6	8.4
FB2	200	91.3	8.9	94.2	4.6
	4000	102.3	12.5	103.3	7.8
	20,000	87.4	18.9	110.8	15.3
FB3	200	98.2	8.6	89.7	7.9
	4000	96.4	9.7	98.4	12.3
	20,000	92.3	5.6	107.6	9.7
HFB1	200	90.6	10.2	90.4	6.3
	4000	91.7	8.1	97.3	5.7
	20,000	94.6	15.9	112.6	10.7
HFB2	200	106.4	17.2	87.6	12.8
	4000	94.6	10.1	90.7	6.3
	20,000	82.6	15.9	106.5	7.7
HFB3	200	115.8	14.0	88.4	10.5
	4000	107.9	11.5	100.2	4.8
	20,000	96.6	8.9	95.5	14.5

**Table 3:** Mass spectrometry parameters of fumonisins, hydrolyzed fumonisins, and their 13C isotope-labeled internal standards.

Analytes	Precursor Ion (*m*/*z*)	Product Ion (*m*/*z*)	Cone Voltage (V)	Collision Energy (eV)
FB1	722.3	352.3 *, 334.3	80	40.34
FB2	706.3	336.3 *, 318.4	80	34.38
FB3	706.3	336.3 *, 318.4	20	32.36
^13^C_34_-FB1	756.3	356.4 *, 738.5	50	43.56
^13^C_34_-FB2	740.4	358.3 *, 722.4	50	53.42
^13^C_34_-FB3	740.4	358.3 *, 376.4	70	53.47
HFB1	406.3	352.3 *, 370.4	10	22.18
HFB2	390.3	336.2 *, 354.3	10	28.22
HFB3	390.3	336.2 *, 354.3	10	28.22
^13^C_22_-HFB1	428.3	374.3 *, 392.3	10	20.20
^13^C_22_-HFB2	412.3	356.3 *, 376.3	10	28.22
^13^C_22_-HFB3	412.3	358.3 *, 376.3	10	28.22

* Quantification product ion.

## Data Availability

Not applicable.

## Supplementary Materials

The following are available online at https://www.mdpi.com/article/10.3390/toxins14020131/s1, Figure S1: The LC-MS/MS chromatograms of the FB1, FB2, FB3, pHFB1a+b, pHFB2a+b, pHFB3a+b, HFB1, HFB2, and HFB3 in the hydrolysis reaction, Figure S2: The LC-MS/MS chromatograms of the analytes in a real sample before (a) and after (b) hydrolysis.
