# Supplementary material for: LC-MS/MS Analysis of Fumonisin B1, B2, B3, and Their Hydrolyzed Metabolites in Broiler Chicken Feed and Excreta
Source: Toxins (Basel). 2022 Feb 9;14(2):131. doi: 10.3390/toxins14020131 (PMC8875997; doi:10.3390/toxins14020131)
Supplement: Supplementary file 1 [file toxins-14-00131-s001.zip › toxins-1516541-supplementary.pdf]

LC-MS/MS Analysis of Fumonisin B1, B2, B3, and Their Hydrolyzed Metabolites in Broiler Chicken Feed and Excreta

Shuo Zhang, Shuang Zhou, Song Yu, Yunfeng Zhao, Yongning Wu and Aibo Wu

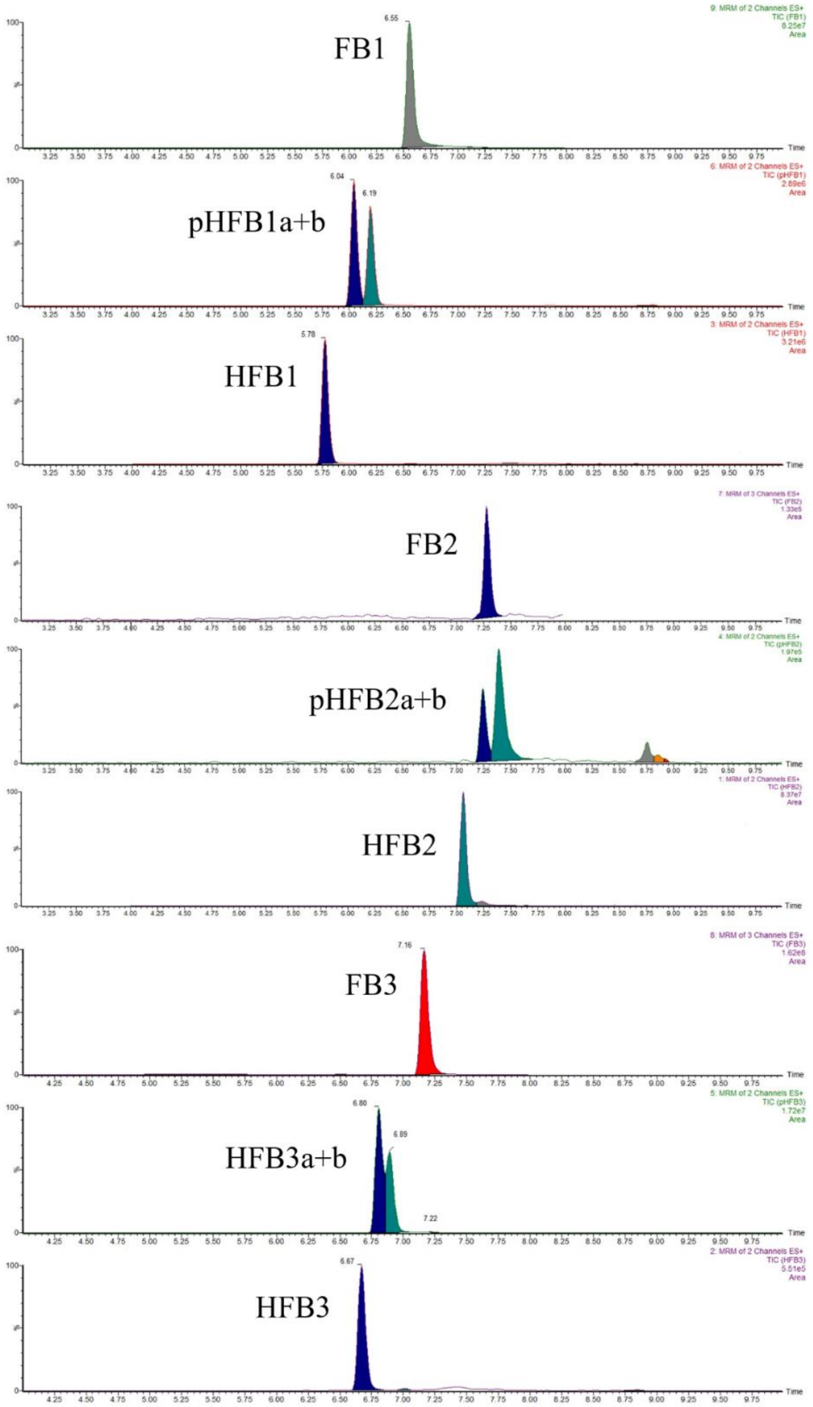

Figure S1. The LC-MS/MS chromatograms the FB1, FB2, FB3, pHFB1a+b, pHFB2a+b, pHFB3a+b, HFB1, HFB2, and HFB3in the hydrolysis reaction.

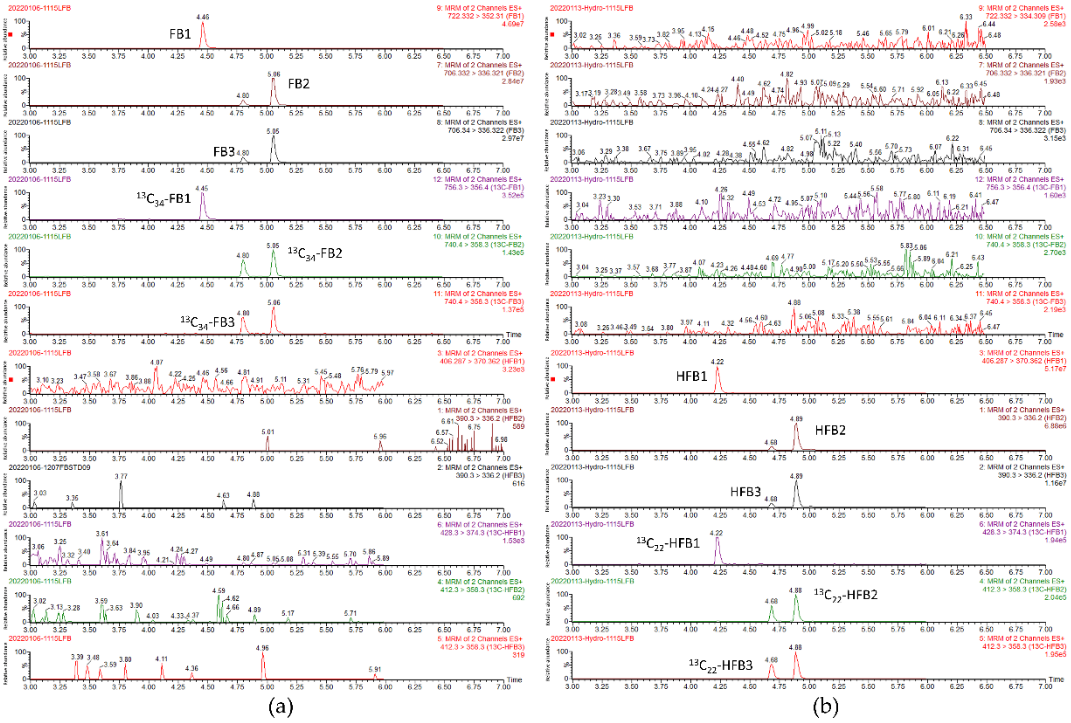

Figure S2. The LC-MS/MS chromatograms of the analytes in a real sample before (**a**) and after (**b**) hydrolysis.
